# Therapeutic Efficacy of Medicinal Plants with Allopathic Medicine in Musculoskeletal Diseases

**DOI:** 10.26502/ijpaes.4490170

**Published:** 2024-12-23

**Authors:** Resmi Rajalekshmi, Devendra K. Agrawal

**Affiliations:** Department of Translational Research, College of Osteopathic Medicine of the Pacific, Western University of Health Sciences, Pomona, California, USA

**Keywords:** Allopathic medicine, Analgesics, Anti-inflammatory drugs, Antioxidants, Complementary alternative therapy, Chondropathy, Herbal drugs, Integrated therapy, Medicinal plants, Musculoskeletal diseases, Osteoarthritis, Osteopathy, Oxidative stress

## Abstract

Musculoskeletal diseases encompass a diverse array of disorders affecting the muscles, bones, joints, and connective tissues, leading to significant impairments in mobility, function, and quality of life. Affecting over 1.3 billion individuals globally, musculoskeletal diseases represent a major source of disability and economic burden. Conventional treatment modalities, including pharmacological interventions and surgical procedures, are frequently limited by adverse side effects, prolonged recovery periods, and patient dissatisfaction, particularly when focused solely on symptom management. In response, complementary and alternative medicine, particularly the use of medicinal plants, has garnered increasing interest to enhance the management of musculoskeletal diseases. Medicinal plants possess a wide spectrum of pharmacologically active compounds with anti-inflammatory, analgesic, and antioxidant properties, making them promising adjuncts to conventional therapies. This review critically evaluates the potential synergy between medicinal plants and allopathic medicine for the management of musculoskeletal diseases, with an emphasis on integrated therapy that combines both modalities. Specifically, a critical discussion is presented on how medicinal plants with scientifically supported pharmacological properties can augment the therapeutic efficacy of conventional medications, reduce their doses, and mitigate adverse effects. Furthermore, the challenges associated with incorporating herbal medicine into established healthcare systems are discussed, including the need for rigorous clinical validation, standardization, and regulatory frameworks. Overall, the article underscores the potential of integrated therapeutic approaches to improve clinical outcomes, enhance patient well-being, and establish a more sustainable model for the treatment of musculoskeletal diseases.

## 1. Introduction

Musculoskeletal diseases encompass a broad spectrum of disorders that affect the muscles, bones, joints, ligaments, and tendons, impairing movement and functionality [1]. The Global Burden of Diseases, Injuries, and Risk Factors Study classifies musculoskeletal diseases into five primary conditions: rheumatoid arthritis, osteoarthritis, low back pain, neck pain, and gout. In addition, a residual category encompasses a wide range of other acute and chronic disorders that affect the locomotor and connective tissue systems [2]. This group includes spondyloarthropathies, inflammatory arthritis (excluding rheumatoid arthritis), vasculitis, autoimmune conditions such as systemic lupus erythematosus, chronic pain syndromes like fibromyalgia, osteopathies, chondropathies, and disorders of bone density, tendons, synovium, and other connective tissues [3]. Musculoskeletal diseases are characterized by symptoms such as pain, stiffness, swelling, and limited mobility, which can significantly impair daily functioning. The underlying causes of these conditions include injury, chronic overuse, genetic predisposition, and autoimmune processes, with the severity ranging from acute and transient to chronic and progressive.

Musculoskeletal conditions are the primary cause of disability and physician visits in the United States, affecting approximately half of the population with activity limitations. These conditions, which include injuries and deformities, significantly impact daily functioning and child development. Globally, musculoskeletal disorders affected 1.3 billion individuals in 2017, resulting in 121,300 deaths and contributing to 138.7 million disability-adjusted life years [4]. In addition to their health impact, these disorders lead to work disability, decreased productivity, and increased healthcare costs. Musculoskeletal injuries, often resulting from falls, accidents, and sports, are particularly prevalent among the elderly and frequently require long-term care. In the United States, the economic burden of musculoskeletal conditions amounted to $380.9 billion in healthcare expenditures, while in Europe, the combined cost of healthcare and productivity losses was €240 billion [5,6].

While conventional allopathic medicine remains the primary approach for treating musculoskeletal diseases, it has several limitations. Pharmacological treatments, such as non-steroidal anti-inflammatory drugs (NSAIDs), corticosteroids, and disease-modifying antirheumatic drugs (DMARDs), are widely used to manage pain and inflammation [7]. However, long-term use of these medications often leads to serious side effects, such as gastrointestinal bleeding, liver or kidney damage, and an increased risk of infections. Surgical interventions, another option for severe cases like joint replacements, can be costly and are associated with long recovery periods, complications, and limited access in low-resource settings. Furthermore, many patients with chronic musculoskeletal disorders report dissatisfaction with conventional care, as it may focus primarily on symptom management rather than addressing the underlying cause or improving overall well-being.

As a result, there has been a growing interest in complementary alternative medicine (CAM) to supplement or replace conventional treatments. CAM therapies include a variety of non-pharmacological approaches such as acupuncture, yoga, chiropractic care, and the use of herbal remedies [8]. These therapies are often perceived as more natural and holistic, aiming to promote physical and mental health while minimizing adverse side effects. Medicinal plants have attracted significant interest for their therapeutic potential in managing musculoskeletal diseases, providing anti-inflammatory, pain-relieving, and antioxidant benefits [9]. The rising popularity of CAM is also driven by patient preference for treatments that align with personal beliefs, cultural practices, and the desire for more sustainable healthcare solutions.

The objective of this review is to explore the potential synergy between medicinal plants and allopathic medicine in the management of musculoskeletal diseases. Integrated therapy—combining conventional treatments with complementary herbal interventions—represents a promising strategy to address the limitations of each approach when used in isolation. Specifically, this review aims to investigate how medicinal plants with scientifically validated pharmacological properties can enhance the effectiveness of allopathic medications, reduce their required dosages, and mitigate side effects. For example, herbal extracts with anti-inflammatory properties could be used alongside NSAIDs to improve pain relief while reducing the risk of gastrointestinal damage.

In this article, there is also a critical discussion evaluating the challenges and opportunities in implementing integrated therapeutic approaches in clinical practice. By identifying relevant studies this review will assess the evidence supporting the co-administration of herbal and conventional treatments. Furthermore, it will discuss the regulatory and standardization challenges of integrating herbal medicine into mainstream healthcare, emphasizing the need for rigorous clinical validation and collaboration between healthcare providers. The goal is to provide insights into how integrated approaches can improve patient outcomes, enhance quality of life, and create a more sustainable model for managing musculoskeletal diseases.

## 2. Musculoskeletal Diseases: Mechanisms, Symptoms, and Current Treatment Approaches

**Figure 1:**
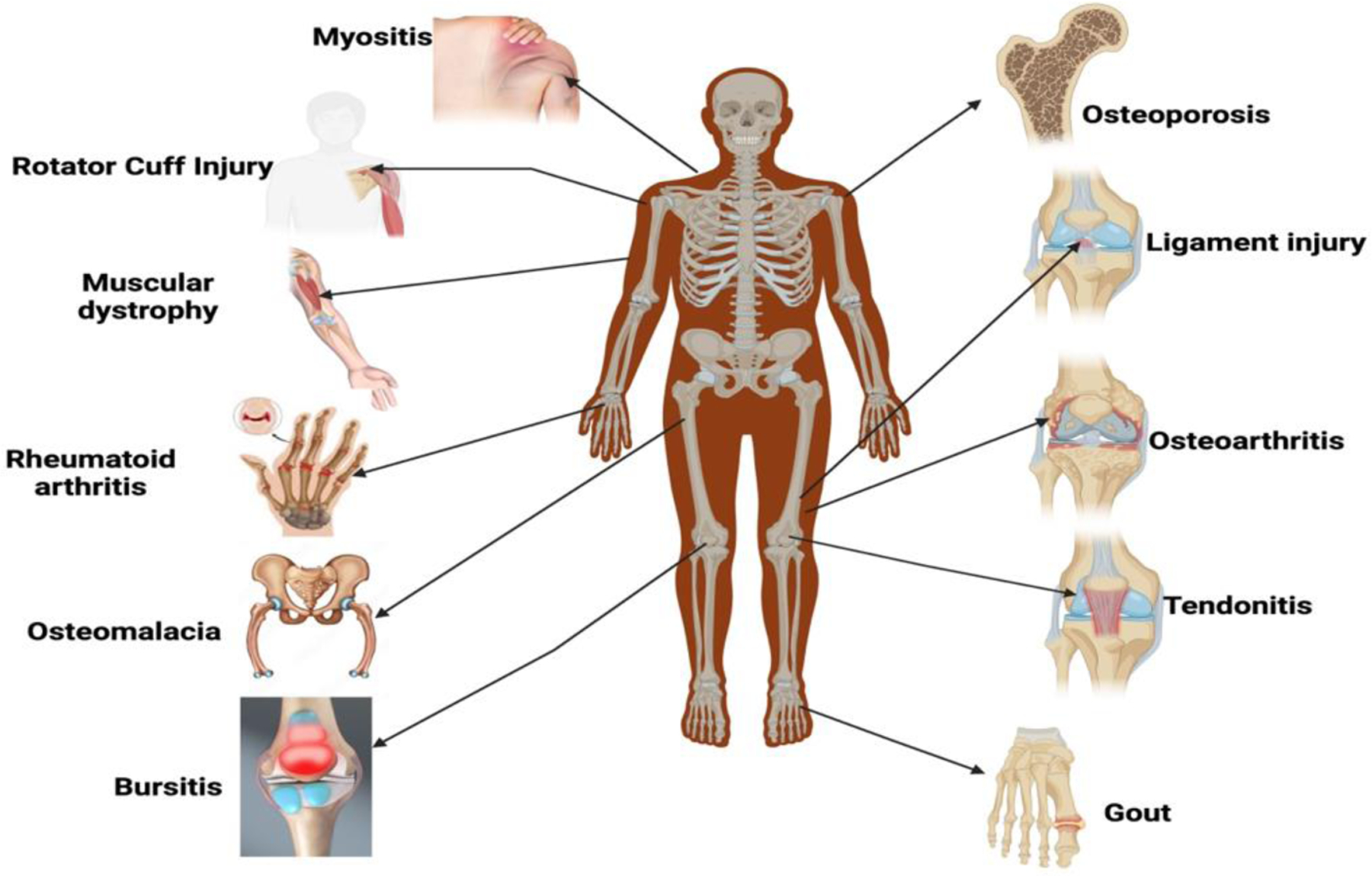
Classification of musculoskeletal diseases.

Musculoskeletal diseases encompass a broad range of disorders affecting bones, joints, muscles, and surrounding tissues (Figure 1). These conditions can result from genetic predispositions, immune system dysfunction, metabolic abnormalities, trauma, or degenerative changes associated with aging. Such diseases often lead to significant pain, disability, and a reduction in quality of life.

### 2.1 Joint diseases

Joint Diseases are among the most common musculoskeletal conditions, often resulting in pain, limited mobility, and a decline in quality of life. One of the primary joint diseases is Osteoarthritis (OA), a degenerative disorder characterized by the gradual breakdown of articular cartilage—the tissue that cushions bones within the joint [10]. The degeneration process is influenced by factors such as mechanical stress, age-related wear and tear, and biochemical changes within the joint environment. As the cartilage thins, the joint space narrows, causing bones to rub directly against each other, leading to the formation of osteophytes, or bone spurs [11]. In some cases, synovial inflammation plays a role in disease progression, with cytokines and enzymes accelerating cartilage breakdown [12]. Patients with OA commonly experience joint pain, stiffness, and swelling, particularly after prolonged activity or periods of inactivity. The knees, hips, hands, and spine are frequently affected, and symptoms typically worsen over time, gradually limiting the patient’s mobility and daily function.

Another prevalent joint disease, Rheumatoid Arthritis (RA), is an autoimmune disorder where the immune system mistakenly attacks the synovium—the lining of the joints [13]. This chronic inflammation prompts the release of inflammatory cytokines, such as tumor necrosis factor-alpha (TNF-α) and interleukin-6 (IL-6), which drive the proliferation of synovial tissue, forming a destructive structure known as pannus [14,15]. Over time, this abnormal growth can erode the joint, resulting in significant damage. RA typically presents with symmetrical joint pain, swelling, and stiffness, especially in smaller joints like the wrists, fingers, and ankles [16]. Morning stiffness, a hallmark of RA reported by 83% of patients, is linked to disease activity and associated with synovial neutrophils and fibrin in cases lasting over an hour [17]. Without proper management, the chronic inflammation in RA can lead to visible joint deformities, muscle wasting, and systemic symptoms such as fatigue and fever, severely impacting daily life.

Gout, another type of joint disease, occurs due to the buildup of monosodium urate crystals within the joints, often due to hyperuricemia [18]. In this condition, TLR2 and TLR4 detect monosodium urate crystals, triggering inflammation through NF-κB and NALP3 inflammasome activation, which leads to IL-1β release [19]. Gout is marked by the abrupt onset of severe pain, most commonly involving the first metatarsophalangeal joint [20]. The affected joint presents with erythema, edema, and hyperthermia, and the pain may endure for several days to weeks. If untreated, chronic gout can lead to the formation of urate crystal deposits, known as tophi, which accumulate beneath the skin and around joints, potentially causing visible deformities and persistent discomfort [21].

### 2.2 Bone diseases

Bone Diseases can severely affect the skeleton’s structural integrity, impacting both mobility and overall health. One of the most common bone disorders is Osteoporosis, a condition marked by decreased bone mass and the deterioration of bone microarchitecture, which increases the risk of fractures. Osteoporosis develops due to an imbalance in bone remodeling, where bone resorption by osteoclasts exceeds bone formation by osteoblast [22]. In osteoporosis, several signaling pathways are involved in the regulation of bone resorption and formation. The RANK/RANKL/OPG pathway is crucial for osteoclastogenesis, where RANKL stimulates osteoclast differentiation and bone resorption, while osteoprotegerin (OPG) acts as a decoy receptor to inhibit this process [23]. Additionally, the Wnt/β-catenin pathway promotes osteoblast differentiation and bone formation, while sclerostin protein inhibits this pathway [24]. Estrogen signaling also regulates bone remodeling, with a decrease in estrogen levels, as seen in postmenopausal women, accelerating osteoclast activity and increasing bone resorption [25]. These interconnected pathways contribute to the imbalance between bone resorption and formation seen in osteoporosis. Osteoporosis is typically asymptomatic until a fracture occurs, commonly affecting the hip, spine, or wrist, with spinal fractures leading to back pain, height loss, and kyphosis [26].

Osteomalacia is a bone disorder marked by softening and weakening of bones due to insufficient mineralization, typically caused by vitamin D deficiency, which disrupts bone mineralization by impeding the vitamin D signaling pathway, where vitamin D binds to its receptor in osteoblasts to regulate calcium and phosphate balance [27]. The fibroblast growth factor 23 (FGF23) pathway, which controls phosphate levels, can exacerbate osteomalacia by reducing phosphate availability [28]. Patients with osteomalacia often present with diffuse bone pain, muscle weakness, and, in severe cases, a waddling gait. The condition may lead to fractures in areas like the spine, pelvis, and legs if left untreated. Osteomalacia is distinct from osteoporosis in its cause (lack of mineralization) and presentation, although both diseases increase fracture risk and impair quality of life [29].

Paget’s disease of bone is a chronic and progressive disorder affecting one or more bones. It is characterized by increased osteoclastic activity, leading to excessive bone resorption, followed by accelerated but disorganized bone formation [30]. The resultant bone has a mosaic pattern of woven and lamellar bone, making it mechanically weak and prone to fractures or deformities.

Osteoclasts in Paget’s disease of bone are abnormally large, increased in number, and may contain viral inclusion-like bodies [31]. The disease is driven by high osteoclastic sensitivity to stimulatory factors such as 1,25 dihydroxy vitamin D and RANKL, with elevated interleukin-6 (IL-6) also contributing to increased osteoclastic activity [32]. While osteoblasts are increased at pagetic sites, they remain morphologically normal and are not considered the primary pathological factor [33].

### 2.3 Muscle diseases

Muscle Diseases encompass a range of disorders characterized by progressive muscle weakness, degeneration, and inflammation, often with genetic or autoimmune origins. Among these, Muscular Dystrophy is a group of inherited diseases that lead to muscle cell deterioration and progressive weakness [34]. Duchenne Muscular Dystrophy (DMD) results from the absence or deficiency of dystrophin, a key component of the dystrophin-glycoprotein complex (DGC) that links the cytoskeleton of muscle fibers to the extracellular matrix [35]. The loss of dystrophin impairs the structural and signaling roles of the DGC, leading to muscle degeneration, inflammation, extracellular matrix degradation, fibrosis, and eventual respiratory or cardiac failure [36]. The DGC interacts with various signaling molecules, including calmodulin (CaM), CaM kinase II (CaMKII), and components of the MAPK and PI3K/Akt pathways, which regulate muscle cell survival, repair, and mechano sensing [37,38]. Disruption of these pathways exacerbates muscle fiber damage through increased Ca^2+^ influx, activation of caspases, and dysregulation of ion channels such as stretch-activated channels (SAC) [39–41]. Additionally, proteins like Grb2, laminin, and α1-syntrophin highlight DGC’s role as a signalosome, with downstream effects on pathways like NF-κB, MAPK, and calcineurin/NFAT, which further contribute to DMD pathology [42]. Understanding these signaling disruptions offers potential therapeutic targets to mitigate disease progression.

Myositis represents another category of muscle disease, marked by inflammation often driven by an autoimmune response. Myositis includes several subtypes, such as polymyositis and dermatomyositis, both of which involve chronic inflammation that damages muscle tissue [43]. In these conditions, the immune system erroneously targets muscle fibers, causing fiber necrosis, inflammation, and ultimately muscle weakness. Patients with myositis commonly experience proximal muscle weakness, particularly in the shoulders and hips, resulting in difficulties with activities such as climbing stairs or lifting objects. Dysphagia, or difficulty swallowing, and profound fatigue are also characteristic symptoms [44]. Dermatomyositis, a subtype, is distinguished by a distinctive rash that can appear on the face, chest, and hands, adding a dermatologic component to the muscular symptoms [45]. Dermatomyositis is driven by complement activation and cytokine expression, while polymyositis and inclusion-body myositis feature CD8+ T cells attacking MHC class I-expressing muscle fibers via the perforin pathway [46]. Persistent MHC class I upregulation in these diseases triggers endoplasmic reticulum stress and NF-κB activation, sustaining inflammation [47]. If untreated, myositis can progress, leading to severe muscle weakness and functional disability.

Myasthenia gravis is a rare autoimmune disorder characterized by the production of antibodies targeting nicotinic acetylcholine (ACh) receptors at the neuromuscular junction (NMJ) in skeletal muscles, leading to muscle weakness and rapid fatigue [48]. The exact trigger of this autoimmune reaction is unknown, although genetic factors may play a role. Myasthenia gravis primarily affects voluntary muscles, especially those that control eye movements, facial expression, and swallowing [49]. Symptoms include drooping eyelids (ptosis), double vision (diplopia), difficulty swallowing, and generalized muscle weakness that worsens with activity.

### 2.4 Tendon and ligament diseases

Tendon and Ligament Diseases involve the connective tissues critical for joint stability and function. Tendinitis refers to inflammation of a tendon, typically arising from repetitive strain or overuse, which causes microtears in the tendon fibers [50]. These microtears induce an inflammatory response, resulting in pain and swelling. Tendinitis commonly affects the shoulder (rotator cuff), elbow (tennis elbow), and Achilles tendon [51].

Rotator cuff injury (RCI) is a prevalent and debilitating musculoskeletal condition, affecting over 20% of the U.S. population and with surgical repair failure rates ranging from 20% to 94% [52]. It is particularly common in individuals over 50 years old, with an estimated incidence of 17% [53]. Risk factors include age, physical activity, diabetes, smoking, body weight, and chronic injuries [54]. RCI significantly impacts quality of life, functional capacity, and occupational performance, causing pain, weakness, limited range of motion, and muscle atrophy. Tears often result from acute trauma, chronic overuse, or age-related degeneration, leading to disruptions in the rotator cuff tendons, which are crucial for stabilizing the glenohumeral joint and enabling shoulder movements [55]. The underlying pathogenesis involves inflammation, extracellular matrix disorganization, inflammasome activation, fatty infiltration, and local immunological factors, contributing to the clinical symptoms and impairment experienced by affected individuals [56–59]. RCI significantly impacts daily living, causing pain, limited movement, muscle atrophy, and financial and psychological distress, particularly among working-age individuals.

A ligament injury is damage to the strong connective tissue that connects bones and stabilizes joints. These injuries often result from trauma, repetitive use, or sudden twisting, leading to overstretching or tearing of the ligament [60]. The most prevalent ligament injuries manifest as sprains, characterized by the stretching or partial tearing of the ligament, as well as complete tears, where the ligament is entirely ruptured [61]. Common sites of ligament injury include the knee—particularly the anterior cruciate ligament (ACL)—the ankle, and the wrist [62]. The symptoms associated with ligament injuries generally include pain, swelling, bruising, instability, and a limited range of motion. The severity of these injuries can vary significantly, with some cases necessitating surgical intervention, especially in instances of complete tears.

## 3. Allopathic Treatments for Musculoskeletal Diseases

**Figure 2:**
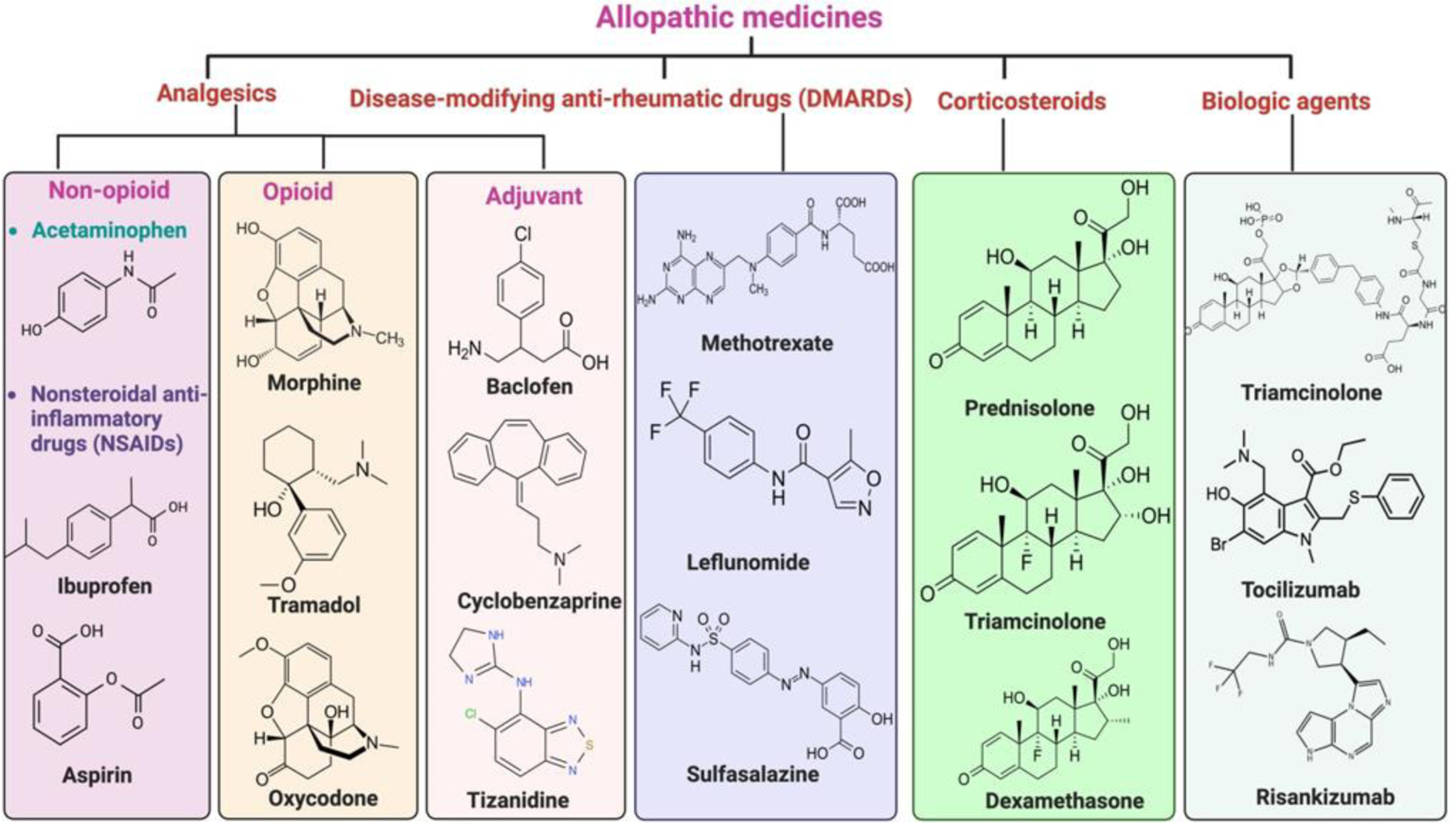
Categories of allopathic therapeutic agents for management of various musculoskeletal diseases.

Treatments include various classes of drugs that aim to relieve pain, reduce inflammation, and, in cases of autoimmune-related musculoskeletal diseases, slow disease progression. While effective in enhancing patients’ quality of life, these pharmacological options carry significant risks of side effects, potential dependency, and limited efficacy for some long-term cases. Different types of allopathic treatments are given in Figure 2.

### 3.1 Analgesics

Analgesics used for pain management are broadly categorized into nonopioid, opioid, and adjuvant medications [63]. Nonopioid analgesics, such as acetaminophen and nonsteroidal anti-inflammatory drugs (NSAIDs), are commonly used for mild to moderate pain [64]. Adjuvant analgesics, although primarily prescribed for conditions other than pain, possess analgesic properties that can be effective in managing specific pain syndromes [65]. Examples include gabapentin, an anticonvulsant; amitriptyline, a tricyclic antidepressant; and various muscle relaxants [66]. Analgesic and musculoskeletal medications are formulated in various delivery systems, including oral tablets and liquids, injectable solutions, inhalable forms, and transdermal patches. Certain pharmaceutical combinations, such as oxycodone with acetaminophen, are designed to enhance pain relief through synergistic effects.

### 3.2 Nonopioid

NSAIDs are commonly used for pain relief, fever reduction, and inflammation control by inhibiting cyclooxygenase (COX) enzymes [67]. Mechanism of action of NSAIDs involves inhibiting the arachidonic acid pathway, preventing the production of inflammatory mediators such as prostaglandins, thromboxanes, and cytokines [68]. This action helps reduce inflammation and pain, but it can impair healing in tissues like tendons and bones. Evidence suggests that NSAIDs, particularly COX-2 inhibitors, may interfere with tissue repair, leading to complications like tendon-to-bone healing issues and delayed recovery after injuries, such as Achilles tendon ruptures [69–72]. However, some studies have shown insufficient evidence to conclusively link NSAID use with impaired recovery, and the choice of NSAID may be crucial.

Recent studies emphasize the importance of balancing the therapeutic effects of NSAIDs with their risks. For instance, while COX-2 inhibitors like celecoxib offer improved GI tolerability compared to non-selective NSAIDs, they have similar cardiovascular risks [73,74]. Long-term NSAID use requires careful monitoring, considering the patient’s age, pre-existing conditions (e.g., ulcers, kidney issues, cardiovascular disease), and concurrent medications to reduce adverse effects. Additionally, recent research questions the conventional use of NSAIDs for acute pain, suggesting they may hinder long-term recovery. A multicenter clinical study found that NSAID treatment in patients with low back pain could delay pain resolution, potentially leading to persistent pain. This study highlighted the role of neutrophils in acute pain resolution, showing that NSAIDs may interfere with the healing process, leading to chronic pain. These findings call into question current pain management practices and suggest that NSAID use may be counterproductive in some cases, particularly for conditions like low back pain [75].

Acetaminophen, commonly known as paracetamol in many parts of the world, is another widely used analgesic for musculoskeletal diseases. Acetaminophen is commonly recommended for mild to moderate pain associated with conditions like muscle strains, mild osteoarthritis, and post-operative pain. The analgesic effects of acetaminophen involve multiple mechanisms. A key pathway involves its metabolism to AM404, which activates transient receptor potential vanilloid 1 (TRPV1) and cannabinoid 1 (CB1) receptors, modulating pain and temperature sensation and inhibiting excitatory synaptic transmission in the spinal cord [76]. It also engages the endogenous opioid and serotonergic systems, enhancing descending inhibitory pathways, with serotonin and opioid receptor interactions playing pivotal roles. Additionally, acetaminophen interacts with prostaglandin H2 synthetase (PGHS) at its peroxidase site, reducing prostaglandin synthesis, particularly under conditions of low oxidative stress, which explains its limited anti-inflammatory effects [77]. Collectively, these pathways highlight acetaminophen’s diverse central and peripheral mechanisms, particularly its enhanced efficacy in inflammatory pain states, paving the way for optimized pain management strategies. Paracetamol has limited long-term efficacy in rheumatic and musculoskeletal diseases (RMDs), providing modest pain relief for knee and hip osteoarthritis (e.g., 3.23 points on a 0–100 pain scale with 4 g/day over 3–12 weeks) but lacking high-quality evidence for other conditions such as chronic low back pain, rheumatoid arthritis (RA), and neuropathic pain [78–80]. Although generally considered safe, chronic high-dose use is associated with risks, including increased gastrointestinal (GI) bleeding, slight elevations in systolic blood pressure (2–4 mmHg), and reduced hemoglobin levels (≥1 g/dL in 20.3% of participants on 3 g/day), particularly when combined with NSAIDs [81,82]. These adverse effects are primarily observed in older populations or those with pre-existing conditions, based on observational studies.

### 3.3 Opioids

The management of pain in rheumatologic conditions has traditionally relied on acetaminophen and NSAIDs, but these often provide insufficient relief or are contraindicated due to side effects or comorbidities. Opioids, including minor agents like tramadol and major opioids, have become alternatives for refractory pain, particularly in chronic non-cancer pain [83]. While meta-analyses indicate opioids may offer modest short-term pain relief and functional improvement compared to placebo, their benefits are comparable to non-opioid alternatives like NSAIDs and antidepressants [84].

Studies reveal that reduced κ-opioid receptor (KOR) expression in the synovium of osteoarthritis (OA) patients is associated with heightened inflammation. Activation of KOR suppresses the production of proinflammatory cytokines TNF-α and IL-6 by blocking macrophage M1 polarization and inhibiting NF-κB translocation, effectively reducing knee pain and synovitis in animal models. These findings highlight the potential of KOR-targeted therapies in addressing OA-related pain and inflammation [85]. KOR also plays a protective role in skeletal tissues by mitigating cartilage degeneration and improving joint lubrication. In KOR-null mice, cartilage breakdown occurs more rapidly after injury. Conversely, KOR activation promotes the expression of anabolic enzymes and prevents degradation through cAMP/CREB signaling pathways. KOR agonists, such as dynorphin, demonstrate dual therapeutic benefits by managing OA pain and protecting against early joint damage [86]. In specific conditions such as rheumatoid arthritis (RA), weak opioids show some short-term efficacy, but no significant long-term advantages have been demonstrated in OA or other rheumatic diseases [87]. Early opioid use in inflammatory conditions may also delay or reduce the initiation of disease-modifying therapies like DMARDs [88].

Opioid therapy is associated with significant risks, including nausea, dizziness, constipation, and higher rates of fractures and hospitalizations for overdose [89]. Long-term use (≥6 months) does not provide superior pain relief or functional benefits compared to non-opioid treatments but increases the risks of dependence, discontinuation, and mortality [90]. Given the modest benefits and substantial risks, current evidence advises against routine opioid use for chronic pain management in inflammatory rheumatic diseases, with safer alternatives being preferred.

### 3.4 Adjuvant analgesics

Adjuvant analgesics, originally developed for non-pain-related purposes, have become effective options for managing pain in various conditions [91]. Muscle relaxants such as baclofen, cyclobenzaprine, and tizanidine are particularly useful for alleviating muscle spasticity and associated symptoms in conditions like multiple sclerosis and spinal cord injuries [92]. Baclofen works by inhibiting reflexes at the spinal level to relieve muscle spasms, pain, and stiffness. It is safe for patients aged 12 years and older but requires caution in those with impaired renal function due to its renal excretion. Common side effects include drowsiness, dizziness, nausea, and muscle weakness, and abrupt discontinuation may result in severe withdrawal symptoms like hallucinations and seizures [93]. Cyclobenzaprine, structurally similar to tricyclic antidepressants, acts at the brainstem to reduce tonic somatic muscle activity, effectively treating acute muscle spasms [94]. It is generally safe for patients aged 15 and older but should be used cautiously in older adults, those with hepatic impairment, and individuals taking CNS depressants. Notable side effects include drowsiness, dry mouth, and dizziness, with a potential risk of serotonin syndrome if combined with serotonergic drugs or MAO inhibitors [95]. Tizanidine, an alpha-adrenergic agonist, reduces muscle spasticity by increasing presynaptic inhibition of motor neurons. It is suitable for adults, though elderly patients may require dosage adjustments. Common side effects include somnolence, dry mouth, dizziness, and the potential for hepatotoxicity, necessitating liver function monitoring [96]. Across all three medications, patient education is essential to optimize therapeutic outcomes and minimize risks, particularly regarding the avoidance of alcohol and CNS depressants, as well as precautions to prevent orthostatic hypotension and other adverse effects.

### 3.5 Corticosteroids

Corticosteroids, such as prednisone and methylprednisolone, are powerful anti-inflammatory medications commonly used in the management of musculoskeletal diseases [97]. These drugs work by modulating the immune system and suppressing the production of inflammatory molecules, offering significant relief in conditions where inflammation plays a central role in pain and tissue damage. Corticosteroids are particularly useful for treating acute flare-ups in inflammatory conditions like tendinitis [98], bursitis [99], and osteoarthritis [100]. They can be administered in various forms, including oral tablets, intravenous injections, or directly into the affected joint or soft tissue through intra-articular injections. The ability to deliver corticosteroids locally via injections makes them especially effective in managing localized inflammation and pain, providing rapid relief.

Glucocorticoid (GC) signaling, primarily mediated by the glucocorticoid receptor (GR), plays a crucial role in modulating inflammation and maintaining cartilage and bone homeostasis in OA. GCs suppress pro-inflammatory cytokines like TNF-α, IL-6, and IL-1β by inhibiting transcription factors such as NF-κB and AP-1 [101,102]. GR activation also promotes anti-inflammatory mediators like DUSP1, GILZ, and ANXA1, which inhibit MAPK signaling, reduce leukocyte migration, and resolve inflammation [103–105]. Intra-articular GC injections, such as dexamethasone, further reduce inflammation and cartilage damage by targeting these pathways.

GR signaling plays a key role in cartilage and bone homeostasis. At physiological levels, glucocorticoids (GCs) maintain extracellular matrix (ECM) integrity by inhibiting matrix metalloproteinases (MMPs) and promoting ECM synthesis, including proteoglycans and type II collagen. Excessive GC exposure disrupts this balance, leading to cartilage degradation, reduced chondrocyte proliferation, and apoptosis by increasing aggrecanase activity and decreasing protective molecules like TIMP1 [106,107]. In subchondral bone, GCs impair osteoblast activity and angiogenesis through VEGF and Wnt/β-catenin pathways [108,109]. These effects contribute to glucocorticoid-induced osteoporosis (GIO) and steroid-induced arthropathy. In the synovium, GCs reduce inflammation by suppressing prostaglandin E2 (PGE2) and TNF-α via 11β-HSD1, which activates cortisone [110,111]. The dose-dependent effects of GR signaling require careful management for optimal OA treatment.

Frequent use of intra-articular corticosteroid injections—while providing rapid symptom relief—can lead to localized risks over time. Repeated injections into joints can weaken the surrounding tissues, including tendons, ligaments, and cartilage. In some cases, this can contribute to further joint deterioration and increase the risk of joint instability [112]. Another critical drawback of corticosteroids is their impact on the body’s endocrine system. Prolonged corticosteroid use can disrupt natural hormone production, especially by inhibiting the function of the adrenal glands. This can lead to adrenal insufficiency, a condition in which the body cannot produce adequate amounts of cortisol, a hormone necessary for stress response and overall metabolic balance [113]. To avoid withdrawal symptoms and adrenal crisis, corticosteroid therapy often needs to be tapered gradually rather than stopped abruptly.

In managing long-term corticosteroid therapy, healthcare providers must carefully balance the benefits and risks. While corticosteroids can provide rapid and effective symptom relief, especially in inflammatory musculoskeletal diseases, their use must be monitored closely to mitigate side effects. For patients requiring prolonged treatment, low-dose corticosteroid regimens may be used, and other adjunct therapies, such as bisphosphonates to protect bones or additional anti-inflammatory medications, may be considered to minimize risks.

### 3.6 Disease-modifying anti-rheumatic drugs (DMARDs)

DMARDs are fundamental treatment for chronic inflammatory and autoimmune musculoskeletal disorders, particularly rheumatoid arthritis (RA), psoriatic arthritis, and ankylosing spondylitis [114]. DMARDs work by targeting and modifying the underlying immune processes that contribute to the inflammatory damage of joints and tissues [115]. Unlike nonsteroidal anti-inflammatory drugs (NSAIDs) and analgesics, which primarily alleviate symptoms like pain and inflammation, DMARDs aim to slow or halt the progression of the disease, preventing long-term joint damage and improving function. As a result, DMARDs are essential for managing autoimmune-related musculoskeletal diseases, where the body’s immune system mistakenly attacks its own tissues, leading to chronic inflammation and potential joint destruction.

Methotrexate and hydroxychloroquine are two of the most prescribed DMARDs in the treatment of autoimmune musculoskeletal diseases. Methotrexate, originally developed as a chemotherapy drug, is widely used for RA [116], as well as other autoimmune conditions like psoriatic arthritis [117] and lupus [118]. Methotrexate works by inhibiting folate metabolism, which suppresses the activity of immune cells that drive inflammation in autoimmune diseases [119]. It is often considered the first-line treatment for RA due to its ability to slow disease progression, reduce inflammation, and prevent joint damage. Hydroxychloroquine, initially used for malaria, is frequently prescribed for conditions such as lupus and rheumatoid arthritis [120]. It functions by modulating the immune system, particularly influencing T cell activity and the production of inflammatory cytokines, thereby alleviating symptoms and reducing the risk of disease exacerbations [121].

One of the primary advantages of DMARDs is their ability to modify the course of autoimmune diseases, preventing joint damage, improving functional outcomes, and decreasing disability over time. These drugs reduce the need for steroids, which have significant side effects when used long-term. By slowing disease progression, DMARDs can improve patients’ quality of life, reducing the frequency and severity of disease flare-ups and minimizing the potential for permanent joint destruction. In RA, for example, the use of DMARDs has been shown to reduce the risk of deformities, preserve joint function, and improve overall mobility [122].

Despite their benefits, DMARDs have several drawbacks, including a slow onset of action, requiring weeks to months to show effects, unlike corticosteroids or NSAIDs [123]. This delay can be challenging for patients with significant pain and inflammation, necessitating additional medications. DMARDs also suppress the immune system, increasing the risk of infections, including respiratory infections and more serious conditions like tuberculosis [124].

### 3.7 Biologic agents

Biologic agents have revolutionized the treatment of musculoskeletal diseases, particularly inflammatory and autoimmune conditions like rheumatoid arthritis, psoriatic arthritis, and ankylosing spondylitis. These therapies are designed to target specific components of the immune system, offering a more targeted approach than traditional DMARDs. TNF-α inhibitors, including monoclonal antibodies (adalimumab, infliximab, golimumab, certolizumab pegol) [125] and the TNFR2-Fc fusion protein (etanercept) [126], are widely used for rheumatoid arthritis (RA), ankylosing spondylitis (axSpA), and psoriatic arthritis (PsA). Anti-IL-6R Abs (tocilizumab, sarilumab) are effective in RA, especially in combination with methotrexate, but lack efficacy in axSpA or PsA [127–130].

Abatacept (CTLA-4-Ig) is effective in RA [131] and PsA [132], and while it suppresses joint destruction, its effect on psoriasis symptoms in PsA is limited. Anti-IL-17A Abs (secukinumab, ixekizumab) [133,134] and the anti-IL-17 receptor Ab (brodalumab) show efficacy in axSpA [135] and PsA [136], with evidence suggesting they slow radiographic progression. Anti-IL-23/IL-12 (ustekinumab) and anti-IL-23 p19 (guselkumab, risankizumab) Abs are effective in PsA but not axSpA or RA, likely due to differing pathophysiological mechanisms [137,138].

However, biologics carry notable risks, including increased susceptibility to serious infections (e.g., tuberculosis and pneumonia) due to immunosuppression, necessitating screening and close monitoring [139]. Potential long-term cancer risks, such as lymphoma and skin cancers, remain a concern. Their high cost limits accessibility, often reserving use for severe cases, while the need for regular injections or infusions adds logistical challenges and risks of localized injection-site reactions [123]. Despite these limitations, biologics offer significant benefits for managing autoimmune musculoskeletal diseases.

## 4. Role of Medicinal Plants in Musculoskeletal Health

**Figure 3:**
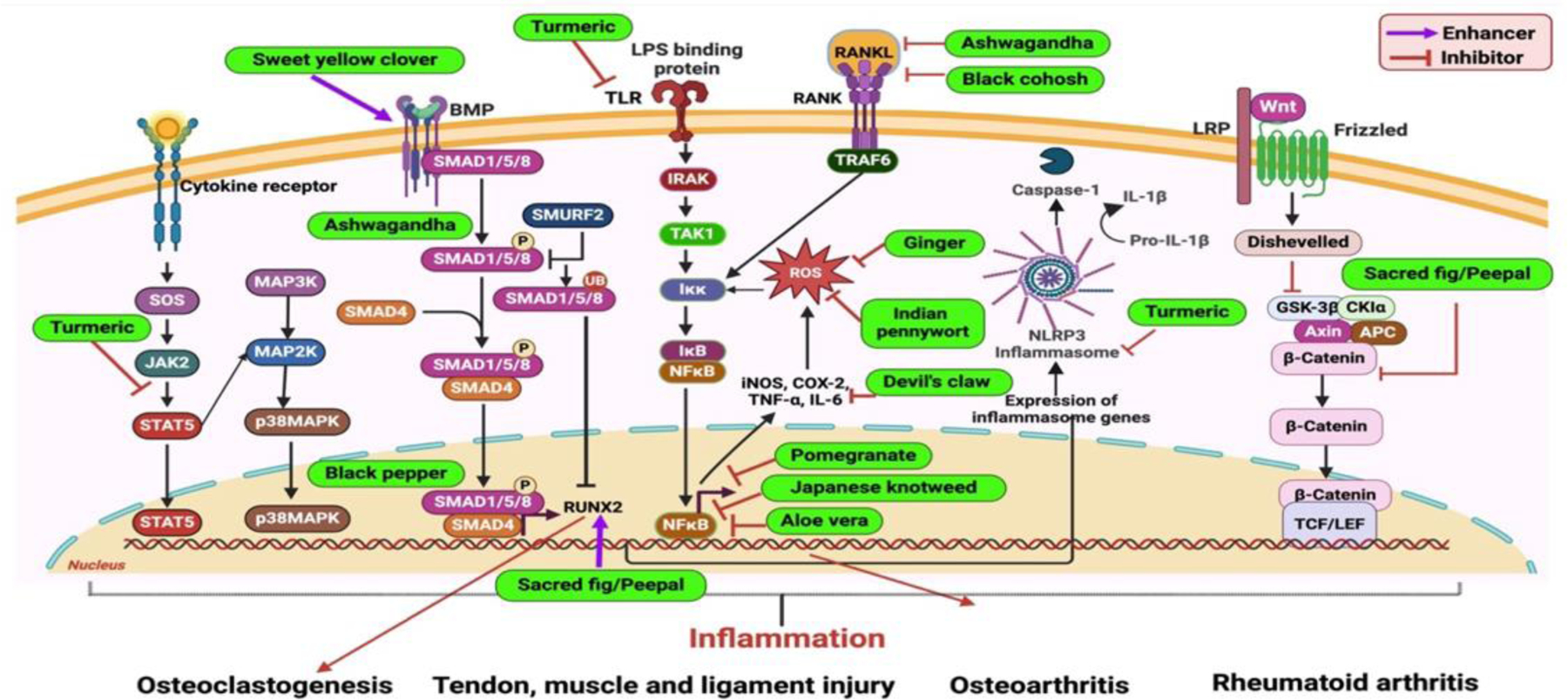
Mechanisms of action of herbal plants in the management of musculoskeletal diseases.

Medicinal plants provide a natural and integrative approach to managing musculoskeletal health, addressing several limitations associated with conventional allopathic treatments. Unlike synthetic drugs, which are often associated with significant side effects such as gastrointestinal disturbances, increased infection susceptibility, and potential long-term toxicity, medicinal plants offer therapeutic benefits with reduced adverse effects [140]. Their bioactive compounds exert anti-inflammatory, analgesic, and tissue-regenerative properties, often acting through multi-targeted mechanisms that address both symptoms and underlying pathologies [141,142]. Additionally, medicinal plants represent a cost-effective and sustainable alternative, particularly in settings where access to expensive pharmaceuticals is limited [143]. This makes them a valuable adjunct or alternative in the management of musculoskeletal disorders, supporting safer and potentially more holistic long-term care. Figure 3 illustrates the mechanisms of action of various medicinal plants in the treatment/management of musculoskeletal diseases.

### 4.1 Anti-inflammatory, analgesics and antioxidant

Medicinal plants have long been used to manage inflammation, pain, and oxidative stress, which are central to many musculoskeletal diseases. These plants contain a wide variety of bioactive compounds, such as flavonoids, alkaloids, terpenoids, and phenolic acids, that exert anti-inflammatory and analgesic effects through various signaling pathways.

Medicinal plants such as Turmeric (*Curcuma longa*), Ginger (*Zingiber officinale*), Long pepper (*Piper longum),* Aloe vera (*Aloe barbadensis*), Liquorice (*Glycyrrhiza glabra*) etc. have demonstrated strong anti-inflammatory properties. The active compound Curcumin in turmeric reduces inflammation by modulating TLRs and key pathways like NF-κB, MAPK and JAK/STAT, and inhibiting the NLRP3 inflammasome [144–147]. It lowers pro-inflammatory mediators such as IL-1 and TNF-α and rebalances the Th17/Treg ratio to control immune responses [148,149].

Ginger contains gingerols and shogaols that inhibit pro-inflammatory cytokines such as TNF-α (Tumor Necrosis Factor-alpha) and IL-6 (Interleukin-6), as well as the COX-2 pathway [150–152]. *Piper longum*, a medicinal plant from the Piperaceae family, contains major compounds like piperlongumine [153], an alkaloid, which reduces inflammatory markers such as TNF-α, IL-6, and IL-23 [154], while also inhibiting ROS production and the JNK/NF-kB signaling pathways in rheumatoid arthritis models [155,156]*. Aloe barbadensis* (Aloe vera) contains phytochemicals such as anthraquinones, cinnamic acid, and aloe emodin [157], which have been shown to inhibit inflammatory markers like IL-1, IL-6, IL-8, TNF-α, and NF-kB [158,159]*. Glycyrrhiza glabra* (licorice), a plant from the Fabaceae family, exhibits anti-inflammatory and anti-arthritic properties due to bioactive compounds like licochalcone A, glycyrol, glycyrrhetinic acid, and glycyrrhizin [160]. Licochalcone A reduces chondrocyte pyroptosis and inhibits pro-inflammatory mediators like IL-1β, IL-18, COX-2, PGE2, iNOS, and NO, demonstrating therapeutic potential in osteoarthritis and antigen-induced arthritis models [161,162]. Glycyrol shows efficacy in collagen-induced arthritis by suppressing inflammatory cytokines and preventing cartilage and bone erosion, but potential harmful effects necessitate careful dosage optimization for therapeutic use [163].

The analgesic properties of several medicinal plants make them valuable for managing pain in musculoskeletal disorders. Willow bark (*Salix alba*) has been used for medicinal purposes for centuries, with salicin (SA), its primary active compound, being the precursor to aspirin [164]. SA has been shown to counteract TNF-α-induced cartilage degeneration, inhibit chondrocyte apoptosis, and promote cell proliferation. It reduces ER stress by blocking IRE1α phosphorylation through the IRE1α-IκBα-p65 signaling pathway. In a rat OA model, intra-articular injection of SA-loaded PLGA significantly protected against OA progression by inhibiting IRE1α-mediated ER stress [165]. Capsaicin, the compound responsible for the burning sensation in chili peppers (*Capsicum annuum*) [166], induces analgesia through TRPV1 desensitization, calcium influx, and activation of calcium-dependent proteins, reducing receptor function and inhibiting Piezo channel activation [167–169]. It also promotes sensory fiber degeneration via apoptosis and non-apoptotic mechanisms, with both peripheral and supraspinal processes, including GABAergic inhibition and opioid activity, modulating its analgesic effects [170]. Devil’s claw (*Harpagophytum procumbens*), a medicinal plant from the Pedaliaceae family found in southern Africa, contains bioactive compounds such as terpenoids and iridoid glycosides, including harpagoside and harpagide, known for their anti-inflammatory and pain-relieving effects [171]. The plant extract inhibits inflammatory processes by reducing cytokine and PGE₂ release, as well as suppressing COX-2, IL-6, and TNF-α mRNA expression [172].

### 4.2 Plants that promote bone health and regeneration

Several medicinal plants have been identified for their potential in promoting bone health and regeneration. These plants contain bioactive compounds that can enhance bone formation, increase mineral density, and aid in the healing of fractures. Some of the key mechanisms include the regulation of osteoblast and osteoclast activity, modulation of bone mineralization, and the reduction of inflammatory processes that affect bone metabolism. Common plants used in bone health include *Withania somnifera* (Ashwagandha), *Melilotus officinalis (*sweet yellow clover*)*, *Ficus religiosa* (Sacred fig/Peepal), *Actaea racemosa*, (Black cohosh), *Piper nigrum* (Black pepper) etc. These plants have demonstrated efficacy in promoting bone density and supporting the regeneration of bone tissue through a combination of anti-inflammatory, antioxidant, and osteogenic properties.

*Withania somnifera*, is an ancient herb used in Ayurveda to treat inflammation-related disorders. Its health benefits, particularly in inflammation, immune modulation, and arthritis pain reduction, are largely attributed to withaferin A (WA), a potent bioactive withanolide [173]. WA promotes bone regeneration by enhancing osteoblastogenesis and inhibiting osteoclastogenesis [174]. It activates osteoblasts via proteasomal inhibition, upregulating osteogenic genes and transcription factors, while reducing osteoclast formation by decreasing TRAP, RANK, and the OPG/RANKL ratio. WA protects RunX2 and Smad proteins via the Smurf2 pathway and suppresses TNF-α production, supporting bone formation and inflammation regulation [175,176].

*Melilotus officinalis* contains active compounds such as coumarins, flavonoids, and phenolic acids, which exhibit anti-inflammatory and antioxidant properties [177]. These compounds promote bone health and regeneration by enhancing osteoblast activity, reducing osteoclast-mediated bone resorption, and mitigating oxidative stress that impairs bone remodeling. Coumarin effectively restored bone turnover and remodeling under high-glucose conditions by promoting osteoblastogenesis and osteoclastogenesis, enhancing alkaline phosphatase activity, collagen type 1 expression, and RANK-osteoprotegerin signaling, while inhibiting AGE–RAGE interaction [178]. Flavonoids influence bone health by regulating cytokine expression, transcription factors, bone matrix proteins, and signaling pathways such as Wnt/β-catenin, BMP/TGF-β, MAPK, ROS, and NFκB/NFATc-1, thus supporting osteoblast differentiation and inhibiting osteoclast activity [179–183].

*Ficus religiosa* holds significant importance in herbal medicine, with its leaves, bark, seeds, and fruits widely used for medicinal preparations [184]. Phytochemical analysis of all parts of Ficus religiosa has revealed the presence of beneficial phenolic compounds such as tannins and flavonoids, along with phytosterols, amino acids, hydrocarbons, furanocoumarins, volatile compounds, and various other secondary metabolites [185]. Polyphenols, including kaempferol, modulate transcription factors like Runx2 and Osterix, and influence cellular signaling pathways such as MAPK, BMP, OPG/RANKL, and WNT, impacting osteoblast and osteoclast functions. Kaempferol promotes bone formation by enhancing osteogenic markers like ALP activity, collagen synthesis, and Runx2 expression, primarily via activation of the WNT signaling pathway [186].

*Actaea racemosa* belongs to the family *Ranunculaceae* and is known for its active compounds, including triterpene glycosides, flavonoids, and phenolic acids [187]. These compounds contribute to its therapeutic effects, including anti-inflammatory, estrogenic, and bone health-promoting properties. A study on ferulic acid, a type of flavonoid in neonatal rats with dexamethasone-induced osteoporosis showed that doses of 20 and 30 mg/kg increased bone mineral density by 25% and 141.7%, respectively. These doses also reduced alkaline phosphatase and osteocalcin levels, improved bone mechanical properties, and modulated signaling by increasing sirtuin1 (SIRT1) and decreasing nuclear factor kappa-B (NF-κB). Histopathological analysis confirmed enhanced bone mineral density, emphasizing ferulic acid’s protective role against osteoporosis through SIRT1 and NF-κB pathways [188]. 25-acetylcimigenol xylopyranoside (ACCX), a triterpenoid glycoside from black cohosh, effectively blocks osteoclastogenesis induced by RANKL or TNF-α by inhibiting the NF-κB and ERK signaling pathways. In vivo, ACCX also reduces TNF-α-induced bone loss, making it a potential lead for developing new anti-osteoporosis agents [189].

*Piper nigrum* belongs to the *Piperaceae* family. It contains active compounds such as piperine, alkaloids, flavonoids, phenolic compounds, and essential oils, which contribute to its medicinal properties, including anti-inflammatory, antioxidant, and bone health effects [190]. Piperine from inhibits osteoclast formation by suppressing the p38 MAPK pathway, reducing c-Fos and NFATc1 expression. It also disrupts osteoclast actin rings and bone resorption, targeting the p38/NFATc1/c-Fos signaling axis [191].

### 4.3 Cartilage protection and repair

Cartilage degradation is a major hallmark of joint disorders such as OA and RA. This condition arises from inflammation, enzymatic breakdown of extracellular matrix components, and oxidative stress. Medicinal plants, rich in bioactive compounds, offer promising alternatives or adjuncts to conventional therapies by targeting key pathways involved in cartilage protection and repair.

*Boswellia serrata* (Indian frankincense) exhibits significant anti-inflammatory properties by inhibiting mediators such as IL-1β, TNF-α, IFN-γ, NO, PGE2, and NF-κB, as well as suppressing 5-lipoxygenase (5-LOX), a key enzyme in leukotriene synthesis implicated in RA and OA [192–194]. Key bioactive compounds, including boswellic acid and acetyl-11-keto-β-boswellic acid, reduce inflammation-induced cartilage degradation and apoptosis in human chondrocytes while alleviating arthritic symptoms in animal models [195,196]. Clinical studies have shown *B. serrata* extract to improve symptoms in OA patients without evidence of toxicity, making it a promising therapeutic candidate for arthritis management [197].

*Cinnamomum cassia* (Chinese cinnamon or cassia bark), belongs to the *Lauraceae* family. Its bioactive compounds include cinnamaldehyde, coumarin, cinnamic acid, epicatechin, tannins, and linalool. Cinnamaldehyde, a major compound in the extract of *Cinnamomum cassia*, shows anti-inflammatory potential in RA by inhibiting pro-inflammatory cytokines like IL-6, IL-1β, and TNF-α through *in vitro*, *in vivo*, and molecular docking studies [198–200]. Tannic acid (TA), a tannin, exhibits anti-inflammatory and cartilage-protective effects in OA. It inhibits the IL-1β-IL-1R1 interaction by binding directly to IL-1β and reduces inflammatory markers (iNOS, COX-2, IL-6, TNF-α, NO, PGE2) in OA chondrocytes. TA also decreases MMP3, MMP13, ADAMTS4, and ADAMTS5 expression, while promoting collagen type II and aggrecan production. It blocks IL-1β-induced MAPK and NF-κB signaling pathways. In a rat OA model, TA alleviates pain, reduces cartilage degradation, and suppresses inflammation, highlighting its potential as a therapeutic agent in IL-1β-related diseases.

*Epimedium sagittatum* (horny goat weed) of the *Berberidaceae* family supports cartilage repair and protection through its bioactive flavonoid, icariin (ICA) [201]. ICA enhances chondrocyte vitality, extracellular matrix synthesis, and collagen formation while mitigating inflammation by inhibiting the NF-κB/HIF-2α and NLRP3 inflammasome-caspase-1 pathways. By suppressing NF-κB activation, HIF-2α expression, and key inflammatory targets like MMP9 and ADAMTS5, as well as reducing pyroptosis via NLRP3 inhibition [202,203].

*Punica granatum L.* (Pomegranate), belonging to the *Punicaceae* family, is abundant in polyphenols [204]. These include anthocyanins (e.g., delphinidin, cyanidin, pelargonidin) that give the fruit its red hue, and hydrolyzable tannins (e.g., punicalin, punicalagin, ellagic acid), which account for 92% of its antioxidant activity [205]. Cyanidin, a bioactive compound classified as both an anthocyanin and a flavonoid, exhibits strong anti-inflammatory and therapeutic properties [206], particularly in the context of managing OA and RA. In OA, it suppresses inflammatory mediators (NO, PGE2, TNF-α, IL-6), catabolic enzymes (ADAMTS5, MMP13), and cartilage degradation markers (aggrecan, collagen II) via Sirt6 activation and NF-κB pathway inhibition [207]. In RA, cyanidin targets IL-17A/IL-17RA signaling, reducing fibroblast-like synoviocyte (FLS) proliferation, inflammatory markers (cyr61, IL-23, GM-CSF), and JAK/STAT-3 signaling, while activating PIAS3 to suppress STAT-3-driven inflammation. Animal studies confirm cyanidin’s ability to alleviate joint inflammation, cartilage damage, and disease progression, highlighting its potential as a natural therapeutic agent for chronic joint disorders [208].

*Polygonum cuspidatum* (Japanese knotweed), belongs to the *Polygonaceae* family and contains bioactive compounds such as resveratrol, emodin, anthraquinones, polydatin, and quercetin, which provide a range of therapeutic effects. Resveratrol (RSV), a polyphenolic phytoalexin [209], protects against OA by inhibiting IL-1β-induced chondrocyte injury. At 24 μM, RSV reduces inflammation and matrix degradation in OA chondrocytes by decreasing MMP-1, MMP-3, MMP-13, COX-2, and iNOS levels, while enhancing collagen-II and aggrecan production. Additionally, RSV suppresses the NF-κB pathway, demonstrating its therapeutic potential for OA treatment [210].

### 4.4 Tendon, muscle and ligament

Soft tissue injuries are common musculoskeletal injuries affecting muscles, tendons, ligaments, and connective tissues, often occurring in athletes or active individuals [211]. They range from minor strains and sprains to severe tears, ruptures, and contusions, typically causing inflammation, pain, and impaired function, posing a significant concern in orthopedics. Medicinal plants can play a key role in the management of soft tissue injuries due to their anti-inflammatory, analgesic, and tissue-healing properties.

The Bu Zhong Yi Qi (BZYQ) decoction is a traditional Chinese formulation known for its significant effectiveness in alleviating fatigue symptoms [212]. It is a traditional Chinese formula consisting of eight herbs: *Radix Astragali* (Huangqi), *Rhizoma Atractylodis* Macrocephalae (Baizhu), Radix Ginseng (Renshen), Pericarpium Citri Reticulatae (Chenpi), *Radix Glycyrrhizae* (Gancao), *Rhizoma Cimicifugae* (Shengma), *Radix Bupleuri* (Chaihu), and *Radix Angelicae Sinensis* (Danggui) [213]. BZYQD enhances muscle function in myasthenia by improving grip strength, reducing muscle degradation markers (MAFbx, MuRF-1), and lowering pro-inflammatory cytokines (IL-1β, IL-6, TNF-α). It decreases ROS accumulation, increases ATP and mitochondrial membrane potential, and modulates the JAK2/STAT3/AKT pathway, providing potential therapeutic benefits for acquired myasthenia [214].

*Centella asiatica* (Indian pennywort), is a medicinal plant from the *Apiaceae* family, valued for its ability to promote wound healing, improve cognitive function, and treat skin conditions [215]. Its bioactive compounds, including triterpenoids like asiaticoside and madecassoside, flavonoids like quercetin, saponins, and phenolic acids, contribute to its anti-inflammatory, antioxidant, and regenerative properties [216]. Quercetin, a flavonoid with antioxidant and anti-inflammatory effects, protects against oxidative stress in the Achilles tendons of diabetic rats. *In vitro*, quercetin treatment reduced the expression of NADPH oxidase (NOX) genes (Nox1, Nox4), reactive oxygen species (ROS) accumulation, and apoptosis in high-glucose conditions. In vivo, quercetin improved collagen fiber arrangement, reduced inflammatory and degradation markers (IL-6, MMP2), and increased Collagen I levels in diabetic rats [217].

*Calendula officinalis* (Marigold**)** belongs to the *Asteraceae* family. Its bioactive compounds include triterpenoids (e.g., faradiol, oleanolic acid), flavonoids (e.g., quercetin), carotenoids, and saponins, which contribute to its anti-inflammatory, antioxidant, and wound-healing properties [218]. A study investigated the effects of topical Calendula cream on Achilles tendon healing in Wistar rats. The Calendula cream (Cal) group demonstrated increased levels of hydroxyproline and non-collagenous proteins compared to the control group, along with enhanced collagen organization observed through polarization microscopy, indicating that Calendula promotes collagen synthesis and organization during the early stages of tendon healing [219].

## 5. Mechanistic Interplay between Phytotherapy and Allopathic Interventions

Phytotherapy and allopathic interventions both play significant roles in the management of musculoskeletal diseases, and when used together, they can offer complementary effects that enhance therapeutic outcomes. The combination of medicinal plants with allopathic drugs may provide synergistic benefits, helping to improve drug efficacy while potentially minimizing side effects.

### 5.1 Synergistic effects of combining medicinal plants with allopathic drugs

The combination of medicinal plants with allopathic drugs has garnered attention as a promising therapeutic strategy for managing musculoskeletal diseases, such as osteoarthritis, rheumatoid arthritis, and tendon injuries. When used together, these treatments can produce synergistic effects, enhancing the overall efficacy of the therapeutic regimen while minimizing the side effects of allopathic drugs.

A randomized trial of 140 knee OA patients compared the efficacy and safety of a curcuminoid complex (BCM-95) combined with diclofenac versus diclofenac alone over 28 days. The combination therapy showed significantly superior improvements in pain and quality of life on the Knee Injury and OA Outcome Score (KOOS) compared to diclofenac alone. Fewer patients in the combination group required rescue analgesics (3% vs. 17%) or histamine-2 (H-2) blockers (6% vs. 28%). The combination also resulted in fewer adverse effects (13% vs. 38%) and was favored by both patients and physicians in global therapy assessments. The study concluded that curcuminoid complex and diclofenac offer greater pain relief, improved function, better tolerability, and may serve as a better alternative for knee OA management [220].

A systematic review and meta-analysis evaluated the effect of combining herbal medicine with Western medicine on bone mineral density improvement in patients with rheumatoid arthritis (RA). Eighteen randomized controlled trials (RCTs) with 1,491 participants were analyzed. Results showed that the combination of herbal medicine with Western medicine significantly improved bone mineral density compared to western medicine alone, particularly in the lumbar spine (0.04 g/cm², 95% CI: 0.03–0.05) and femoral neck (0.03 g/cm², 95% CI: 0.02–0.03). Commonly used herbal medicines, such as Xianlinggubao and Hanbikang-tang, showed additional benefits when combined with methotrexate. Improvements were also observed in bone markers and inflammatory indicators. While the findings suggest a promising role for herbal medicine and western medicine combination therapy, further high-quality, large-scale clinical trials are needed to confirm these results [221].

Another study evaluated the bone-protective effects of the Chinese herbal formula ELP (Epimedii Herba, Ligustri Lucidi Fructus, and Psoraleae Fructus) in combination with antiresorptive drugs in ovariectomized rats. Co-administration of ELP with raloxifene resulted in significant increases in bone mineral density at the tibia and distal femur and reductions in urinary deoxypyridinoline levels, indicating enhanced bone strength and reduced bone resorption. However, no synergistic effects were observed in groups treated with alendronate, suggesting that ELP selectively enhances raloxifene’s efficacy, potentially allowing for dose reduction [222].

A 90-day double-blind randomized controlled trial assessed the effects of resveratrol as an adjuvant to meloxicam in 110 patients (aged 45–75) with mild to moderate knee OA. Participants received 15 mg/day of meloxicam with either 500 mg/day of resveratrol or a placebo. Pain severity measured using the Visual Analogue Scale-100, and serum inflammatory biomarkers (IL-1β and IL-6, TNF-α, C-reactive protein, and complement proteins C3 and C4) were evaluated. The resveratrol group showed a significant reduction in pain severity and inflammatory biomarkers compared to the placebo group, indicating that resveratrol may be an effective “add-on” therapy for managing pain and inflammation in knee OA [223].

### 5.2 Potential drug-herb interactions and contraindications

The concurrent use of herbal remedies and conventional drugs is common in managing musculoskeletal diseases such as RA and OA. However, these combinations often pose significant risks due to herb–drug interactions, which can influence pharmacokinetics (drug absorption, distribution, metabolism, and excretion) and pharmacodynamics (drug action and effect) [224]. These interactions are particularly concerning given the widespread use of herbal supplements, frequently without proper medical oversight or disclosure to healthcare providers.

Many drugs used in the management of RA and OA, such as methotrexate (MTX), leflunomide, glucocorticoids, NSAIDs, and biologics, are associated with significant risks, including hepatotoxicity, nephrotoxicity, QT prolongation, and interactions involving CYP enzymes or transport proteins [225,226]. These risks are often exacerbated by herbal remedies, which can lead to complex pharmacokinetic and pharmacodynamic interactions.

Hepatotoxic herbs like green tea, ginger, and turmeric may worsen the liver toxicity of drugs such as MTX, tacrolimus, leflunomide, and acetaminophen [227–229]. Certain herbs, including peppermint tea and milk thistle, inhibit CYP enzymes, altering the metabolism of drugs like rifampicin and warfarin [230,231].

Herbs with antiplatelet effects, including ginkgo, bilberry, and evening primrose oil, increase bleeding risks when combined with platelet-inhibiting drugs like NSAIDs [232–234]. Similarly, warfarin’s anticoagulant effect can be either potentiated by antiplatelet or coumarin-containing herbs or counteracted by vitamin K-rich herbs, underscoring the complexity of interactions. Cannabinoids from *Cannabis sativa* may also enhance CNS depression when co-administered with opioids or selective serotonin-norepinephrine reuptake inhibitors (SSNRIs) [235,236]. These findings highlight the need for careful evaluation of herbal-drug interactions to mitigate adverse effects.

Herbs can also significantly impact drug metabolism through CYP enzyme and transport protein modulation. Drugs such as cyclosporine, tacrolimus, and tofacitinib, which are substrates of both CYP3A4 and P-glycoprotein, exhibit increased susceptibility to interactions that affect their pharmacokinetics. Chamomile, garlic, ginger, turmeric, and St. John’s Wort can interfere with these pathways, leading to altered drug levels and increased toxicity or diminished efficacy [237–239]. St. John’s Wort, a well-documented inducer of multiple CYP enzymes and pGP, is particularly problematic, reducing the efficacy of drugs like glucocorticoids, tofacitinib, and tacrolimus, potentially causing therapeutic failure [240,241]

The clinical unpredictability of herb–drug interactions is exacerbated by variations in herbal preparation, concentration, and patient-specific factors such as comorbidities and metabolic capacity [242]. This unpredictability is particularly concerning in patients with hepatic or renal impairments, where drug metabolism and clearance are already compromised. Furthermore, the lack of standardized dosing for herbal products increases the risks of therapeutic failure or toxicity. Mitigating these risks requires comprehensive strategies, including thorough medication reviews, biomarker monitoring (e.g., liver and kidney function tests), and patient education on the potential hazards of herbal supplements. Collaboration between conventional and complementary medicine practitioners can enhance safety and optimize outcomes in integrative care. While herbal remedies offer therapeutic potential, their use alongside conventional drugs demands meticulous oversight. Addressing these risks through robust research, healthcare provider education, and standardized practices is essential to ensuring the efficacy and safety of integrative treatments.

## 6. Challenges and Limitations of Integrative Treatment for Musculoskeletal Diseases

Integrative treatment approaches for musculoskeletal diseases aim to combine conventional medicine with complementary therapies such as herbal medicine, acupuncture, and physical therapies. While these approaches hold promise for improving patient outcomes, several challenges hinder their effective implementation. Key obstacles include the lack of standardization in herbal treatments, limited awareness and acceptance by healthcare practitioners, regulatory barriers, and patient adherence concerns. Addressing these challenges is critical for advancing integrative strategies as a reliable option in the management of musculoskeletal diseases.

### 6.1 Challenges related to the assessment of efficacy

The use of herbal medicines is often questioned due to the lack of scientific evidence supporting their efficacy, with concerns that reliance on herbal remedies may delay access to conventional, evidence-based treatments. Despite the long history of herbal medicine, scientific studies on their efficacy have yielded mixed results.

The debate between traditional herbal knowledge and evidence-based medicine (EBM) is rooted in differing research methodologies. EBM advocates for Randomized Controlled Trials (RCTs) and systematic reviews as the gold standard for proving clinical efficacy [243]. However, many herbal medicine researchers argue that RCTs are not the only valid means of knowledge generation, citing the long history and widespread use of herbal medicines. The cultural differences in research methods, such as the Chinese medicine concepts of Qi and Yin-Yang, may not align with Western medical frameworks but can still provide valuable insights into treatment efficacy [244].

Regulatory authorities like the European Medicines Agency (EMA) and the U.S. Food and Drug Administration (FDA) require rigorous evidence for herbal medicines to gain marketing approval, but even conventional medicines often do not meet the ideal evidence standards, particularly for complex conditions with multiple comorbidities [245–246]. There is also increasing recognition of the limitations of RCTs in addressing the needs of patients with chronic conditions. Conditional marketing authorization, used by both the European Union and FDA, offers an alternative regulatory approach, allowing faster access to essential medicines while ensuring post-market monitoring for safety and efficacy. This model may provide a more balanced approach to evaluating herbal medicines, emphasizing real-world data and long-standing usage rather than strict pre-market clinical trials.

### 6.2 Challenges in quality control of herbal medicines

The safety and efficacy of herbal medicines are inherently linked to the quality of their source materials. Factors such as genetic variations, environmental conditions, agricultural practices, and proper collection methods significantly influence the consistency and integrity of raw materials. Ensuring high-quality raw materials is challenging due to the variability in plant species and cultivation environments. Adherence to Good Agricultural and Collection Practices, accurate species identification, and strict storage and sanitation protocols are essential for maintaining the quality of the raw materials used in herbal formulations [247].

Quality control of finished herbal products is even more difficult, particularly in multi-herb mixtures where verifying the inclusion of all intended plant species is challenging. Unlike conventional pharmaceuticals, herbal medicines often lack standardized protocols to ensure uniformity across batches. Variability in preparation methods, such as teas, tinctures, and extracts, can lead to differences in the concentration and bioavailability of active compounds [248]. For example, curcumin, the active compound in turmeric, has low water solubility, and its bioavailability is enhanced in lipid-based or alcohol-based preparations [249]. Inconsistent preparation techniques among manufacturers can further contribute to unreliable therapeutic outcomes.

Fluctuations in the potency of herbal products also arise from external factors like harvesting time, storage conditions, and processing methods. Improper handling can degrade sensitive bioactive compounds, diminishing their therapeutic effects. Additionally, contamination with harmful substances such as pesticides, heavy metals, and microbial toxins is a significant concern, particularly for patients with compromised organ function [250]. The lack of rigorous quality control standards and regulatory oversight exacerbates these challenges, leading to considerable variability in the safety and effectiveness of herbal products.

### 6.3 Challenges in safety monitoring of herbal medicines

The global increase in the use of herbal products, coupled with weak regulatory frameworks in many countries, has underscored the need for effective safety monitoring systems. Adverse events related to herbal medicines can arise from several factors, including misidentification of plant species, adulteration with undeclared substances, contamination with hazardous materials, overdoses, inappropriate use by consumers or healthcare providers, and interactions with conventional medications [246,251].

Safety monitoring is particularly complicated by the variability in the geographical origin of plant materials, processing methods, and routes of administration. Furthermore, the lack of standardized scientific nomenclature leads to confusion, as plants with different pharmacological properties may share similar common names. For instance, *Heliotropium europaeum*, which contains hepatotoxic alkaloids, can be confused with *Valeriana officinalis*, a plant with sedative properties, due to the overlap in their common names [252]. Effective safety monitoring requires comprehensive documentation of the scientific names, plant parts used, and manufacturing details of herbal medicines. Collaboration among botanists, phytochemists, pharmacologists, and regulatory authorities is essential to develop robust safety evaluation frameworks and ensure consumer protection.

### 6.4 Clinical challenges in combined therapies

The clinical integration of complementary therapies faces significant barriers, starting with limited awareness and acceptance among healthcare practitioners. Many clinicians lack sufficient knowledge of complementary treatments, including their mechanisms, potential benefits, and contraindications [253]. This knowledge gap fosters skepticism and reduces the likelihood of their inclusion in routine care. Without standardized education and training programs, practitioners may also feel unprepared to address potential risks, such as drug-herb interactions or liability concerns.

Regulatory hurdles further complicate the landscape. Unlike pharmaceuticals, herbal products are often not subjected to strict regulations, resulting in variability in quality and efficacy [187,254]. The absence of clear guidelines for combining conventional and complementary treatments creates uncertainties, such as the risk of overlapping toxicities when combining NSAIDs with herbal anti-inflammatories like turmeric [232,254]. Moreover, limited insurance coverage for complementary therapies adds financial barriers, deterring their integration into treatment plans.

Patient adherence is another critical challenge. Integrative therapies often involve complex regimens requiring the simultaneous use of medications, supplements, and lifestyle changes. This complexity, combined with the financial burden and time commitment, can overwhelm patients, leading to poor compliance. Additionally, misinformation from unregulated online sources may prompt self-medication or deviation from evidence-based recommendations, further compromising treatment outcomes.

### 6.5 Addressing the challenges

To realize the potential of integrative treatments for musculoskeletal diseases, a multifaceted approach is essential. Education and training programs for healthcare practitioners should focus on the evidence-based application of complementary therapies, fostering interdisciplinary collaboration and comprehensive care plans. Regulatory reforms are needed to establish quality control standards and harmonized guidelines for combining conventional and complementary treatments. Expanding insurance coverage for evidence-supported therapies can also reduce financial barriers.

For patients, simplifying treatment regimens and providing culturally sensitive educational resources can improve adherence. Engaging patients in shared decision-making and addressing their concerns about complementary therapies can foster trust and encourage commitment to the treatment plan. By addressing these challenges, integrative approaches can offer a more effective, patient-centered strategy for managing musculoskeletal diseases, blending the strengths of conventional and complementary medicine.

## 7. Future Directions and Recommendations

To fully harness the potential of both traditional and modern medicine, fostering interdisciplinary collaboration is imperative. This involves the integration of the extensive knowledge base of traditional herbal practices with the scientific rigor of contemporary clinical research. Encouraging communication and collaboration among researchers, healthcare providers, and experts in traditional medicine will enable a more comprehensive understanding of herbal treatments. Furthermore, large-scale, long-term clinical trials are crucial to evaluate the efficacy and safety of herbal medicines within modern healthcare systems. These trials should encompass diverse populations, considering variables such as age, comorbidities, and concurrent medication use, to ensure the applicability and generalizability of the findings.

The standardization of herbal medicine is an essential step to ensure its safe and effective use. This process should include the establishment of uniform guidelines for the identification, preparation, and dosage of herbal products. Comprehensive testing for purity, potency, and contaminants is necessary to prevent adverse effects and enhance the quality control of herbal medicines. The development of internationally recognized standards will facilitate the integration of herbal remedies into mainstream medical practice, offering consumers safe and reliable treatment options. Efforts should focus on ensuring that herbal products consistently meet safety, quality, and efficacy criteria, thereby reducing the risk of misidentification and misuse.

The creation of clear, evidence-based guidelines for integrative therapy is vital to optimizing the benefits of combining traditional and modern medical approaches. These guidelines should focus on developing personalized treatment protocols for musculoskeletal diseases, accounting for the distinct mechanisms of both conventional therapies and herbal interventions. By tailoring treatment plans to individual needs, medical history, and specific musculoskeletal disease, healthcare providers can enhance patient outcomes. Integrative approaches must also ensure that herbal remedies do not interact negatively with prescribed medications and that their use does not delay access to proven, evidence-based care. Collaborative frameworks involving both conventional and complementary medicine practitioners are necessary for the successful implementation of integrative therapies in clinical settings.

## 8. Conclusion

In conclusion, the integration of medicinal plants in the treatment of musculoskeletal diseases offers complementary benefits, enhancing therapeutic outcomes when combined with conventional treatments. The evidence underscores the importance of personalized medicine through integrative therapy, where individualized treatment plans, based on patient-specific needs, medical history, and the types of musculoskeletal diseases, can optimize both the efficacy and safety of interventions. Moving forward, the development of sustainable and holistic treatment strategies will require ongoing research, collaboration between traditional and modern healthcare practitioners, and the establishment of rigorous standards for the safe and effective use of herbal remedies. This approach will facilitate the creation of comprehensive, evidence-based treatment protocols that harness the full potential of both medicinal plants and modern medicine, ultimately improving patient care and outcomes in the management of musculoskeletal diseases.
